# A Cross-Sectional Study of Fibromyalgia and Post-acute COVID-19 Syndrome (PACS): Could There Be a Relationship?

**DOI:** 10.7759/cureus.42663

**Published:** 2023-07-29

**Authors:** Alaa Akel, Bilal Almanasyeh, Abdulrahman Abo Kobaa, Ahmed Aljabali, Ahmed Al-Abadleh, Asem Alkhalaileh, Abdel Rahman Alwardat, Mohammed Y Sarhan, Mohammad Abu-Jeyyab

**Affiliations:** 1 Orthopaedic Surgery, School of Medicine, Mutah University, Al-Karak, JOR; 2 General Practice, School of Medicine, Mutah University, Al-Karak, JOR; 3 School of Medicine, Jordan University of Science and Technology, Irbid, JOR; 4 School of Medicine, Balqa Applied University, Salt, JOR; 5 Internal Medicine, King Abdullah University Hospital, Irbid, JOR; 6 Orthopedic Surgery, Hashemite University, Al-Zarqaa, JOR; 7 Surgery, School of Medicine, Mutah University, Al-Karak, JOR

**Keywords:** ss, wpi, pacs, covid-19, fibromyalgia

## Abstract

Background

Post-acute COVID-19 syndrome (PACS) is a syndrome characterized by a wide spectrum of symptoms emerging after clearance of coronavirus 2019 (COVID-19) infection. These symptoms include fatigue, myalgia, arthralgia, cognitive dysfunction, and many other psychiatric symptoms. Given that fibromyalgia patients have similar symptoms, we conducted a web-based cross-sectional study to investigate the prevalence and predictors of fibromyalgia patients who recovered from COVID-19.

Methods

Data were collected between the 9th and 19th of March 2022 using a web-based survey. The questionnaire consisted of 25 questions gathering sociodemographic information, comorbid diseases and features of acute COVID-19 infection. Lastly, the American College of Rheumatology (ACR) survey criteria completed the questionnaire.

Results

A final sample of 404 individuals (75% women) filled out the form. Of these, 80 (19.8%) satisfied the ACR survey criteria for fibromyalgia (93.8% women). A multivariate logistic regression model including demographic and clinical factors showed that female gender (OR: 6.557, 95% CI: 2.376 - 18.093, *p* = 0.001) and dyspnea (OR: 1.980, 95% CI: 1.146 - 3.420, *p* = 0.014) were the strongest predictors of being classified as having post-COVID-19 fibromyalgia. Bivariate correlation revealed that age (r = 0.200, *p* = 0.001) and duration of COVID-19 infection (r = 0.121, *p* = 0.015) were directly correlated with fibromyalgia symptom (FS) score.

Conclusion

Our data suggest that clinical features of fibromyalgia are common in patients who recovered from COVID-19 and that dyspnea and female gender increase the risk of developing post-COVID-19 fibromyalgia.

## Introduction

COVID-19

Coronavirus 2019 (COVID-19) infection is a disease that was first discovered in December 2019 in Wuhan, China [1]. It is caused by the infection of an RNA virus called severe acute respiratory syndrome coronavirus 2 (SARS-CoV-2) [1]. The virus is highly contagious to man and therefore was considered a pandemic by WHO in March 2020 following its spread around the world [2]. The disease is multisystemic and ranges in severity from an asymptomatic course in some patients to potentially lethal manifestations in others [3]. Apart from this, a group of symptoms was recognized as a long-term sequela in patients who recovered from COVID-19, and it was called post-acute COVID-19 syndrome (PACS) [4]. On that note, myalgia - the cardinal symptom of fibromyalgia - was reported in about 30% of PACS patients [5].

Fibromyalgia

Fibromyalgia is a chronic disease that is characterized by diffuse musculoskeletal pain [6]. The exact etiology behind the disease is still unknown. However, it is attributed to a disturbance in one’s perception of pain [7]. Various factors have been described as a possible trigger of fibromyalgia, such as major tissue damage e.g., road traffic accidents and surgeries, and major psychological trauma. In addition, the familial clustering of fibromyalgia cases has been reported, proposing a possible genetic link. Several studies have proposed viral infections e.g., human immunodeficiency virus (HIV) and hepatitis C virus (HCV) as potential risk factors for fibromyalgia development [7].

Diagnosis of fibromyalgia

In 1990, the American College of Rheumatologists (ACR) developed criteria for fibromyalgia diagnosis. These criteria are dependent on patient examination, as it relied on eliciting pain by pressure on 18 specific body areas. Having at least 11 tender points out of 18 meant that the patient is labeled as a case of fibromyalgia [8]. Later in 2010, the ACR set new diagnostic criteria that identified fibromyalgia cases based on a combination of two scores: symptom severity (SS) scale and widespread pain index (WPI) [8]. Patients diagnosed with fibromyalgia fulfill one of these two conditions: the first is having a WPI ≥ 7 and an SS scale of ≥ 5, while the second is having a 6 ≥ WPI ≥ 4 and having an SS scale of ≥ 9 [8]. Following that, the 2010 ACR criteria have been used to generate a valid and reliable questionnaire that removed the need for an examiner and diagnosed patients by self-reporting for the purposes of questionnaire-based cross-sectional studies [9].

Fibromyalgia and COVID-19

Symptoms of fibromyalgia have been reported to worsen during COVID-19 infection [10]. However, studies regarding the possible role of COVID-19 as a trigger of fibromyalgia are lacking. Therefore, knowing that COVID-19 is an infectious disease of a viral origin, and its subsequent syndrome is notably associated with myalgia, it only makes sense to assume that there is a possible link between COVID-19 and fibromyalgia development. Thus, this study was conducted to explore the correlation between the two diseases.

## Materials and methods

Study design and setting

This study is a cross-sectional study that was carried out in the period between the 9th and the 19th of March 2022 via Google Forms which is a free tool that is extensively used to make and distribute web-based medical surveys [11]. Ethical approval for this study was retrospectively obtained from the ethics committee at Mutah University, Faculty of Medicine, Karak, Jordan (reference number: 992023. Date: 13 February 2023). The study targeted the general population at both Madaba and Amman governorates, Jordan. Reaching this population was possible through social media platforms and no direct benefit or gain was obtained through voluntarily answering the questionnaire.

Fibromyalgia diagnosis

To classify subjects as either fibromyalgia positive or negative, the ACR survey criteria were used [12]. It is a modification of the ACR 2010 criteria that was made for the purpose of epidemiological and clinical studies [13]. Both WPI and SS scales were calculated for each individual. Subsequently, fibromyalgia symptom (FS) score was calculated through the summation of both WPI and SS scales. Labeling a case as positive for fibromyalgia was achieved whenever the FS score was ≥ 13. This cut-off point was confirmed as the best to separate fibromyalgia-positive patients from negative ones [12].

Survey development

The survey developed by Ursini et al. was adopted in this study [14]. The survey was translated into the Arabic language by an independent native Arabic speaker who is fluent in Italian. The resultant questionnaire was then translated back to Italian by an independent native Italian speaker who is fluent in Arabic and did not read the original questionnaire. Comparison between the original and the back-translated questionnaire was done to ensure that no major changes in meaning occurred. Moreover, a pilot study was conducted on 30 individuals who did not have a role in survey development. Of these, 15 were previously diagnosed with COVID-19 and the other 15 were not. This pilot study assessed the understandability of the questionnaire, and subsequent modifications were made according to participants’ suggestions. Modifications on the survey included the correction of simple grammar mistakes and the addition of a graph that demonstrated all the 19 body parts that participants were asked if they feel pain at. It was found that the survey took an average of five minutes to be completed. All questions were mandatory, and the survey could not be submitted unless the questions were answered. A response was collected after the participant presses “send”.

Survey structure

The survey was composed of three pages with a total of 25 questions that were preceded by an introduction stating the overall goal of the questionnaire, names of researchers and their contact information. In addition, researchers added a statement stating that all data collected will be anonymized and used only for research purposes. As for the questions, the first part (Q1 - Q11) collected sociodemographic features of the participants and their comorbid conditions. The second part (Q12 - Q19) included questions regarding COVID-19 status. Lastly, the third part (Q20 - Q25) resembled the ACR survey criteria for fibromyalgia diagnosis.

Statistical analysis

Continuous variables were expressed in mean ± SD, and categorical variables were expressed in frequencies and percentages. For comparison between continuous variables, Mann-Whitney U test and Student’s t-test were used according to the normality of distribution. As for categorical variables, Chi-square and Fisher's exact tests were used where appropriate to investigate for any significant differences. Moreover, bivariate correlation was used to check for possible correlations between continuous variables and was reported as Pearson correlation coefficient (R). Lastly, univariate and multivariate logistic regression models were built to determine the predictability of different variables to dependent variables, and it was reported by both 95% confidence interval (CI) and odds ratio (OR). P value of ≤ 0.05 was deemed as significant and data analysis was carried out using SPSS version 25 (IBM Corp., Armonk, NY, USA).

## Results

General sociodemographic characteristics

In the mentioned period, a total of 706 people responded to the survey. However, 302 of them had a negative COVID-19 nasopharyngeal swab and thus they were excluded from the study. As illustrated by Table 1, the final sample was composed of 404 participants. With a mean age of 37.6 ± 12 years and a mean BMI of 26.6 ± 3.9 kg/m^2^, the majority of participants were females (75.0%), married (66.8%) and unemployed (52.2%). As for their pre-existing comorbidities, irritable bowel syndrome (IBS) (38.1%) and psychiatric disorders (23%) were most frequently reported. During the course of participants’ COVID-19 infection, fever was the most frequently reported symptom (56.2%), followed by cough (54.7%), myalgia (52.7%) and dyspnea (45.0%). The mean duration of COVID-19 infection was 12.8 ± 5.3 days. Moreover, most patients (89.1%) were treated at home, while only six (1.5%) patients and one (0.2%) needed a ward and an intensive care unit (ICU) admission respectively. As for the medications given, paracetamol was given for every patient who was treated regardless of the treatment setting (367 patients, 90.8%). Low molecular weight heparin (LMWH) and dexamethasone were only given to the seven (1.7%) patients who were admitted to the ward or ICU. Lastly, oxygen supply was only needed for four (1.0%) of the admitted patients.

**Table 1:** General characteristic features of the included sample. LMWH: low molecular weight heparin, IBS: irritable bowel syndrome

	Overall (n = 404)
Age (years)	37.6 ± 12.0
BMI (kg/m^2^)	26.6 ± 3.9
Sex	
Male, n (%)	101 (25)
Female, n (%)	303 (75)
Marital status	
Single, n (%)	134 (33.2)
Married, n (%)	270 (66.8)
Employment status	
Unemployed, n (%)	221 (52.2)
Employed, n (%)	193 (47.8)
COVID-19 duration (days)	12.8 ± 5.3
COVID-19 symptoms	
Fever, n (%)	227 (56.2)
Cough, n (%)	221 (54.7)
Dyspnea, n (%)	182 (45.0)
Headache, n (%)	122 (30.2)
Myalgia, n (%)	213 (52.7)
Treatment setting	
No treatment, n (%)	37 (9.2)
Home treatment, n (%)	360 (89.1)
Hospital admission, n (%)	6 (1.5)
ICU admission, n (%)	1 (0.2)
COVID-19 treatment	
Paracetamol, n (%)	367 (90.8)
Dexamethasone, n (%)	7 (1.7)
LMWH, n (%)	7 (1.7)
Oxygen, n (%)	4 (1.0)
Pre-existent comorbid diseases	
IBS, n (%)	154 (38.1)
Psychiatric disease, n (%)	93 (23.0)
Rheumatological disease, n (%)	60 (14.9)
Neurological disease, n (%)	24 (5.9)
Cancer, n (%)	7 (1.7)
Fibromyalgia status	
Negative, n (%)	324 (80.2)
Positive, n (%)	80 (19.8)

Comparison between fibromyalgia and fibromyalgia-free subjects following COVID-19 infection

As illustrated by Table 2, a total of 80 (19.8%) participants satisfied the ACR survey criteria and were labeled as fibromyalgia positive. Fibromyalgia cases had a significantly higher age (41.0 ± 13.3 years) compared to those who were free of fibromyalgia (36.7 ± 11.5 years) (p = 0.004). In addition, 93.8% of fibromyalgia cases were female, which is significantly higher than the percentage of females who were not diagnosed with fibromyalgia (70.4%) (p = 0.001). Moreover, the percentage of fibromyalgia cases who experienced dyspnea during their COVID-19 infection (57.5%) was significantly higher than those who were not diagnosed with fibromyalgia (42.0%) (p = 0.012). Among fibromyalgia cases, pre-existing comorbid diseases were significantly more prevalent compared to non-fibromyalgia cases. The difference was found to be the greatest regarding psychiatric diseases (36.3% vs 19.8%, p = 0.002), followed by IBS (50% vs 35.2, p = 0.015), rheumatological diseases (22.5% vs 13.0%, p = 0.032) and lastly followed by neurological diseases (11.3% vs 4.6%, p = 0.025). Neither of the groups significantly differed regarding cancer prevalence.

**Table 2:** Comparison between fibromyalgia and non-fibromyalgia subjects. LMWH: low molecular weight heparin, IBS: irritable bowel syndrome

	Fibromyalgia negative (n = 324)	Fibromyalgia positive (n = 80)	P value
Age (years)	36.7 ± 11.5	41.0 ± 13.3	0.004
BMI (kg/m^2^)	26.5 ± 3.8	26.9 ± 4.0	0.451
Sex			0.001
Male, n (%)	96 (29.6)	5 (6.3)	
Female, n (%)	228 (70.4)	75 (93.8)	
Marital status			0.887
Single, n (%)	108 (33.3)	26 (32.5)	
Married, n (%)	216 (66.7)	54 (67.5)	
Employment status			0.148
Unemployed, n (%)	175 (54.0)	36 (45.0)	
Employed, n (%)	149 (46.0)	44 (55.0)	
COVID-19 duration (days)	12.6 ± 5.3	13.7 ± 5.1	0.094
COVID-19 symptoms			
Fever, n (%)	179 (55.2)	48 (60.0)	0.443
Cough, n (%)	178 (54.9)	43 (53.8)	0.848
Dyspnea, n (%)	136 (42.0)	46 (57.5)	0.012
Headache, n (%)	101 (31.2)	21 (26.3)	0.390
Myalgia, n (%)	170 (52.5)	43 (53.8)	0.837
Treatment setting			0.591
No treatment, n (%)	32 (9.9)	5 (6.3)	
Home treatment, n (%)	287 (88.6)	73 (91.2)	
Hospital admission, n (%)	4 (1.2)	2 (2.5)	
ICU admission, n (%)	1 (0.3)	0 (0.0)	
COVID-19 treatment			
Paracetamol, n (%)	292 (90.1)	75 (93.8)	0.314
Dexamethasone, n (%)	5 (1.5)	2 (2.5)	0.557
LMWH, n (%)	5 (1.5)	2 (2.5)	0.557
Oxygen, n (%)	3 (0.9)	1 (1.3)	0.793
Pre-existent comorbid diseases			
IBS, n (%)	114 (35.2)	40 (50.0)	0.015
Psychiatric disease, n (%)	64 (19.8)	29 (36.3)	0.002
Rheumatological disease, n (%)	42 (13.0)	18 (22.5)	0.032
Neurological disease, n (%)	15 (4.6)	9 (11.3)	0.025
Cancer, n (%)	4 (1.2)	3 (3.8)	0.123

Predictors of fibromyalgia after COVID-19 infection

Bivariate correlation revealed that both age (r = 0.200, p = 0.001) and duration of COVID-19 infection (r = 0.121, p = 0.015) were directly correlated with FS score (Table 3). Moreover, to further explore the effect of other variables in predicting the fibromyalgia outcome, logistic regression model was built. As shown by Table 4, univariate logistic regression proposed participants’ age (OR: 1.029, 95% CI: 1.008 - 1.052, p = 0.008), female gender (OR: 6.316, 95% CI: 2.477 - 16.106, p = 0.001), the occurrence of dyspnea during COVID-19 infection (OR: 1.870, 95% CI: 1.140 - 3.069, p = 0.013), pre-existing IBS (OR: 1.842, 95% CI: 1.124 - 3.019, p = 0.015), pre-existing rheumatological disease (OR: 1.949, 95% CI 1.052 - 3.612, p = 0.034), pre-existing neurological disease (OR: 2.611, 95% CI: 1.099 - 6.206, p = 0.030) and pre-existing psychiatric disease (OR: 2.310, 95% CI: 1.358 - 3.931, p = 0.002) as risk factors for fibromyalgia. Additionally, multivariate logistic regression was conducted to eliminate the effect of confiding factors. The results revealed that female gender (OR: 6.557, 95% CI: 2.376 - 18.093, p = 0.001) and dyspnea (OR: 1.980, 95% CI: 1.146 - 3.420, p = 0.014) were independent risk factors for fibromyalgia. Interestingly, in multivariate analysis, being married was found to be a protective factor against the development of fibromyalgia (OR: 0.462, 95% CI: 0.232 - 0.920, p = 0.028). However, its effect was not significant in univariate analysis.

**Table 3:** Pearson correlation between fibromyalgia symptom (FS) score and continuous variables.

	FS score	
	r	P value
Age	0.200	0.001
BMI	0.050	0.320
Time since COVID-19 (months)	0.074	0.137
In-COVID-19 duration (days)	0.121	0.015

**Table 4:** Univariate and multivariate regression model. LMWH: low molecular weight heparin, IBS: irritable bowel syndrome

	Univariate			Multivariate		
	OR	95% Cl	P value	OR	95% Cl	P value
Age	1.029	1.008 - 1.052	0.008	1.029	0.998 - 1.061	0.071
BMI	1.024	0.962 - 1.091	0.450	1.023	0.941 - 1.112	0.600
Being female	6.316	2.477 - 16.106	0.001	6.557	2.376 - 18.093	0.001
Being married	1.038	0.616 - 1.750	0.887	0.462	0.232 - 0.920	0.028
Being employed	1.435	0.878 - 2.347	0.150	1.632	0.941 - 2.831	0.081
COVID-19 duration	1.041	0.993 - 1.090	0.095	1.030	0.979 - 1.084	0.255
COVID-19 symptoms						
Fever	1.215	0.738 - 1.999	0.443	1.194	0.682 - 2.093	0.535
Cough	0.953	0.583 - 1.557	0.848	0.951	0.547 - 1.651	0.857
Dyspnea	1.870	1.140 - 3.069	0.013	1.980	1.146 - 3.420	0.014
Headache	0.786	0.453 - 1.363	0.391	0.829	0.449 - 1.533	0.550
Myalgia	1.053	0.645 - 1.720	0.837	1.012	0.578 - 1.772	0.967
COVID-19 treatment						
Paracetamol	1.644	0.619 - 4.363	0.318	0.673	0.218 - 2.071	0.490
Dexamethasone	1.636	0.312 - 8.590	0.561	2.834	0.175 - 45.819	0.463
LMWH	1.636	0.312 - 8.590	0.561	2.834	0.175 - 45.819	0.463
Oxygen	1.354	0.139 - 13.196	0.794	0.711	0.020 - 25.657	0.852
Pre-existent comorbid diseases						
IBS	1.842	1.124 - 3.019	0.015	1.646	0.947 - 2.861	0.077
Psychiatric disease	2.310	1.358 - 3.931	0.002	1.782	0.977 - 3.250	0.060
Rheumatological disease	1.949	1.052 - 3.612	0.034	1.211	0.588 - 2.492	0.603
Neurological disease	2.611	1.099 - 6.206	0.030	1.595	0.584 - 4.360	0.363
Cancer	3.117	0.683 - 14.215	0.142	2.471	0.433 - 14.093	0.308

## Discussion

Fibromyalgia is a chronic condition that results in widespread musculoskeletal pain. It is frequently associated with fatigue, sleep disturbances, psychological distress, and cognitive impairment [15]. Therefore, fibromyalgia significantly impairs the quality of life of affected individuals [15]. Reports have been conflicting regarding fibromyalgia prevalence, as it varied from 0.4% in some reports to 11% in others [16]. However, Wolfe et al. have attributed this difference to selection bias [16]. Up to this day, the exact etiology behind fibromyalgia is unclear. However, several studies reported that fibromyalgia was significantly more common among patients infected with human immunodeficiency virus (HIV) and hepatitis B and C viruses, compared to their corresponding healthy controls. Therefore, it was hypothesized that viral infection plays a role in fibromyalgia development [7,17,18]. Fatigue and myalgia, the cardinal symptoms of fibromyalgia, have been extensively reported in patients following their COVID-19 infection [19]. This fact, along with clinical observations, attracted the authors’ interest into investigating the relationship between COVID-19 infection and the development of fibromyalgia. Hence, this cross-sectional study was conducted.

We successfully managed to diagnose about 20% of the studied sample with fibromyalgia. This is in accordance with the results of Savin et al., as they reported that 15% of their included sample developed fibromyalgia after being discharged following recovery from COVID-19 infection [20]. The 5% difference between the two studies could be explained by the difference in data collection methods, as we utilized a web-based questionnaire while Savin et al. collected patients’ responses via an interview [20]. In our study, female gender was found to be an independent risk factor for developing fibromyalgia. This finding is consistent with many studies, including Savin et al. [19-21]. On the contrary, however, Ursini et al. found that male gender is independently associated with a 10-fold increase risk of developing fibromyalgia [14]. This finding may be supported by the prestigious study conducted by Wolfe et al., in which they demonstrated that fibromyalgia is not as female-dominant as it is commonly thought to be, and the high female:male ratio is a result of selection bias [16]. However, this evidence may not be enough to conclude the predominance of males in fibromyalgia and, therefore, further investigation is needed.

Despite the lack of our study for an accurate measurement method for COVID-19 severity, results have shown that the patients who suffered dyspnea during their COVID-19 infection - which is a sign of severity in COVID-19 [22] - were at a higher risk to develop fibromyalgia after recovery. Interestingly, being married was a protective factor against developing fibromyalgia. For cultural reasons, only married women in the Middle East are allowed to get pregnant due to prohibition of sexual intercourse out of wedlock. Thus, married women are more likely to experience higher levels of progesterone - due to pregnancy and subsequent lactation - which is protective against pain severity in fibromyalgia [23,24]. Moreover, our results have shown that neither hospitalization nor the need for supplemental oxygen were significantly correlated with the development of fibromyalgia. This was concordant with the results of Savin et al. [20]. However, Ursini et al. stated that there was a significantly higher prevalence of fibromyalgia among those who required oxygen supplementation during their COVID-19 infection [14]. These contradictory results could be viewed as a consequence of the low sample size of hospitalized patients in the three studies. To our knowledge, we are the first in the Middle East and North Africa to assess the relationship between COVID-19 infection and the development of fibromyalgia. However, the absence of a control group could be viewed as a potential limitation for this study.

Several unanswered questions must be dealt with soon. First, do some COVID-19 variants have a higher tendency to cause fibromyalgia than others? Second, will the conventional method of fibromyalgia treatment be effective in treating post-COVID-19 fibromyalgia? Lastly, do SARS-CoV-2 vaccines provide prophylaxis against such a disease? In conclusion, being female and experiencing dyspnea during the acute phase of COVID-19 infection were observed to significantly increase the likelihood of developing post-COVID-19 fibromyalgia.

## Conclusions

This study investigated the prevalence and predictors of fibromyalgia in patients who recovered from COVID-19 using a web-based survey. The results showed that 19.8% of the participants met the criteria for fibromyalgia and that female gender and dyspnea were the strongest risk factors. The study also found that age and duration of COVID-19 infection were positively correlated with fibromyalgia symptom score. These findings suggest that COVID-19 may trigger or exacerbate fibromyalgia in some patients and that screening and management of fibromyalgia symptoms should be considered in post-COVID-19 care.
